# Maximum entropy spectral analysis for circadian rhythms: theory, history and practice

**DOI:** 10.1186/1740-3391-11-6

**Published:** 2013-07-11

**Authors:** Harold B Dowse

**Affiliations:** 1 Department of Mathematics and Statistics, School of Biology and Ecology, 5751 Murray Hall, University of Maine, Orono ME 04469, USA

## Abstract

There is an array of numerical techniques available to estimate the period of circadian and other biological rhythms. Criteria for choosing a method include accuracy of period measurement, resolution of signal embedded in noise or of multiple periodicities, and sensitivity to the presence of weak rhythms and robustness in the presence of stochastic noise. Maximum Entropy Spectral Analysis (MESA) has proven itself excellent in all regards. The MESA algorithm fits an autoregressive model to the data and extracts the spectrum from its coefficients. Entropy in this context refers to “ignorance” of the data and since this is formally maximized, no unwarranted assumptions are made. Computationally, the coefficients are calculated efficiently by solution of the Yule-Walker equations in an iterative algorithm. MESA is compared here to other common techniques. It is normal to remove high frequency noise from time series using digital filters before analysis. The Butterworth filter is demonstrated here and a danger inherent in multiple filtering passes is discussed.

## Background

Physiological processes in almost all plants and animals have adapted to the cycles in the environment, be they daily (circadian), tidal, lunar, synodic lunar monthly or annual [1]. Oscillatory behavior with periods of less than 24-h, termed ultradian, are also commonly found, occasionally embedded in circadian or other rhythms [2]. This adaptation to cycles in the environment has occurred through the evolution of a biological timekeeper, a true temperature-compensated oscillator providing temporal information at all levels of physiology and behavior [1]. Thorough investigation of these oscillators requires that the periodic evolution of the processes in time be characterized precisely as to the length of the periods seen, as these are the manifestation of the clock process [3]. In addition, the relative robustness and regularity of the rhythms is of considerable interest. Numerical samplings of any process that evolves in time, taken at appropriate intervals, form time series, the stuff and substance of biological rhythm research.

Analysis of time series may be simply done. In early work by Bünning on bean plant leaf movements, periodicity was estimated by measuring peak-to-peak intervals on chymographs that registered leaf position [4]. Analysis technique has progressed considerably since that time and now offers an array of possibilities for estimates of period length [5]. This paper deals with a very useful method called Maximum Entropy Spectral Analysis, or MESA, developed by John Parker Burg in the 1960s in answer to shortcomings of the principal analysis technique up to that time, Fourier analysis [6-8]. We will first discuss Fourier analysis, noting the problems that MESA was developed to fix and how they can be circumvented with MESA. We will pay attention to the theoretical underpinnings so that this popular method will not be a “black box” and will show the basics of how the spectrum is computed. Given that the biologist necessarily works with time series that are either inherently irregular or contain major trends, tools that can ameliorate these problems when used in conjunction with MESA will be introduced and examples of their benefits discussed.

### Biological rhythm data

Circadian rhythms are studied in systems ranging from intracellular fluorescence to complex behaviors such as running wheel activity; data acquisition and format vary accordingly. For example, when studying the activity of an enzyme, the variable may be continuous and an appropriate sampling interval must be chosen. This must be rapid enough to avoid “aliasing” in the periodicity region of interest. This occurs when the sampling interval is longer than the period being recorded and is famously seen in old western movies when the spokes of wagon wheels seem to be going backwards; sampling frequency must be no less than twice the frequency of the cyclic process of interest. This constraint is the Nyquist or foldover frequency [9]. Faster sampling is normal, however, to ensure no detail is lost and that accurate period estimates result. There are two important things to consider. The first, is resolution, which is the ability to separate two frequencies as being distinct, for example a 24-h circadian peak with a 24.8-h lunar daily peak. This is equivalent to optical resolution, in which two objects in an image can be separable [10]. Resolution is theoretically limited by the number of cycles in the data set, or in optics, by the diameter of the lens. A completely separate problem is the ability to discern a periodic signal in noise. This is sensitivity, and both are important.

Biological rhythm data are commonly not continuous and consist of unary events unlike the record left on a kymograph by a bean plant. Here, other constraints begin to play a role. Running wheel activity in mammals and the breaking of an infrared light beam by *Drosophila* are useful examples. Here, individual events occur, and are summed across arbitrary intervals or “bins”. Bin size affects the output of time series analysis and this effect can be profound when bin size is too small (Review: [11]). Bin sizes of 10 min up to an hour are common in rhythms work.

**Figure 1:**
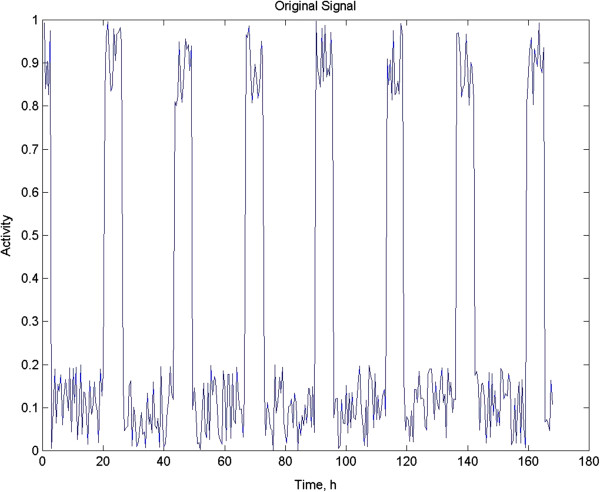
**An artificially produced time series with an arbitrary maximum amplitude of one.** It is a square wave with 20% white noise added. The power in the series is: 0.62.

For the purposes of illustration of the techniques being discussed, we will analyze a simulated data set. This is useful in this sort of discussion, as one may know precisely what the parameters of the signal are. The series considered here is a 23-h square wave with 20% white noise added consisting of 336 values produced at half hour intervals for 7 days using our own software. I chose a square wave as it is important to show that any analysis be effective for waveforms deviating markedly from the badly overused sinusoid. A simple time plot of the data is shown in Figure 1.

### The autocovariance and autocorrelation functions

Given a particular signal, even if it appears clearly rhythmic in a simple time plot, it is important that an objective statistical test be employed to determine if significant periodicity is present. Such a robust test is autocorrelation analysis [5]. In this analysis, the time series is initially lined up with itself in register and correlation analysis is applied yielding the coefficient, r. In this case, no matter what the signal looks like, correspondence is one to one and r = 1. The two series are then set out of register or “lagged” by one interval. The result is a decrement in r. The drop depends on the series; if it is a noiseless sinusoid, the change will initially be small, but if it is white noise, the drop will be very large, since the value of any given point has no relation whatsoever to any other point either near or far in time. Lagging proceeds one interval at a time up to about N/3. The process is usually limited to this point since the power of the test is reduced with the decrement of each pair of lags off the ends of the series. r values are plotted as a function of the lag yielding the autocorrelogram function. In a rhythmic series, r will continue to decline, becoming negative and reaching a minimum when the peaks and valleys in the two series are out of phase by one half cycle. A second positive peak will occur when the peaks and valleys are back in phase, but one cycle out of register. The envelope of decay of the autocorrelation peaks is a function of the regularity in the series and this can provide a useful way of characterizing the regularity in the signal, as will be discussed below (Reviews: [5,12]).

When computing the correlation coefficient, the output is normalized by dividing by the variance of the complete data set, but this need not be so and the output is then “covariance”, or the autocovariance function [5]. Autocorrelation is commonly employed, as it allows comparisons among wide-ranging experiments.

The autocorrelation function also yields a valuable way to quantify the regularity of the signal both in terms of variation in period and the presence of noise. The height of the third peak in this function, counting the peak at lag zero as one, is taken as the Rhythmicity Index, or RI. This value relies on the decay of the envelope of the function and is normally distributed so it may be used in statistical analyses (review: [12]). The RI of the test signal is 0.697.

**Figure 2:**
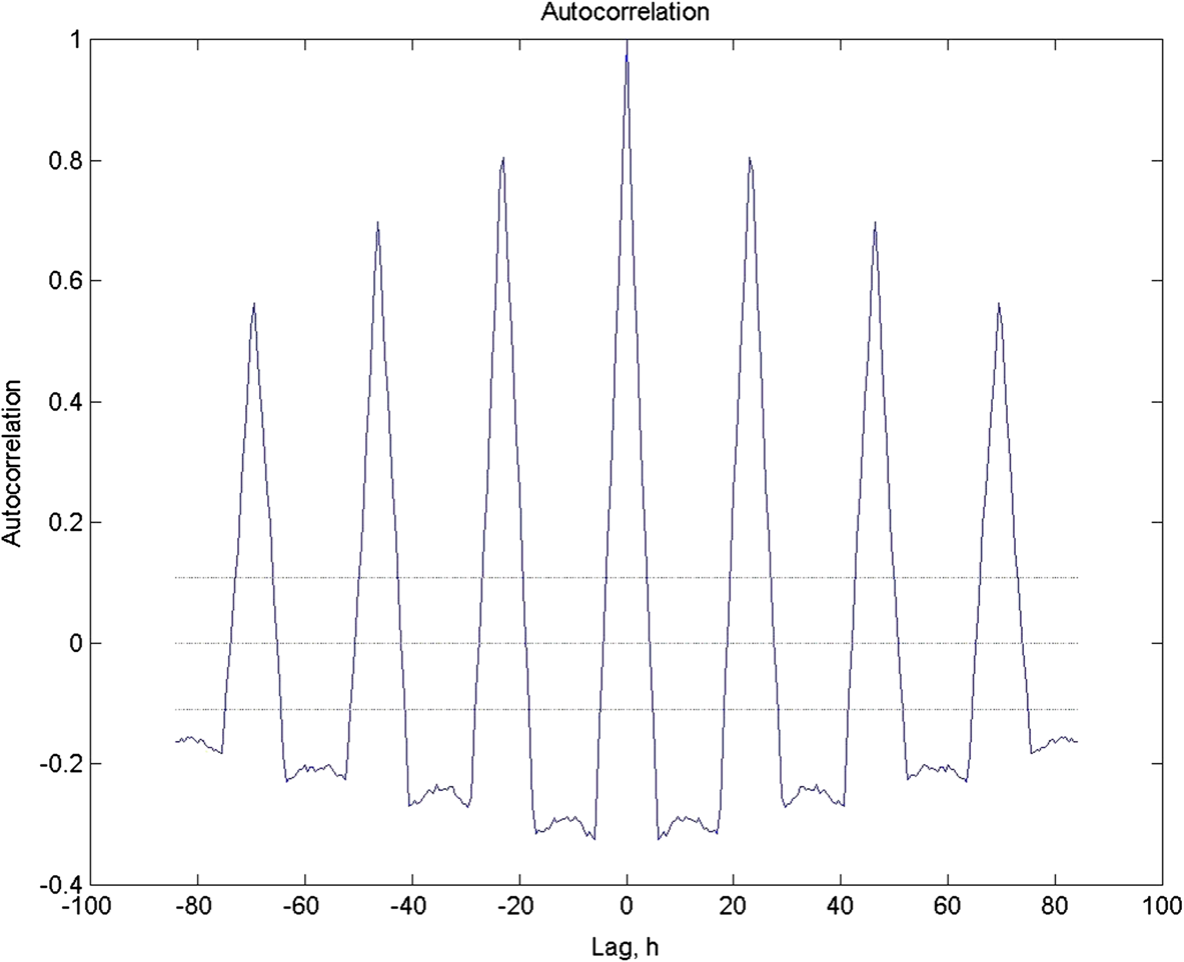
**This is the autocorrelation of the data depicted in Figure**1**.** Note the height of the third peak, which is the RI and equals 0.697.

It is useful to have a formal criterion for the significance of rhythmicity in data. The 95% confidence interval for testing the significance of a given peak in the autocorrelogram is 2/√N [5]. Plus and minus confidence intervals are commonly plotted as flat lines, the decrement in N as values are lost being ignored (see above discussion). Repeated peaks equaling or exceeding the confidence interval are usually taken to imply significant rhythmicity, but there is room for subjective interpretation [5,12]. Figure 2 shows the autocorrelation function for the test data set depicted in Figure 1. Since the lagging can be done in either direction, Lag 0, where r = 1 is at the center and the function is mirrored to the left as well. This view can make visual interpretation easier.

### Fourier analysis

Beginning in the late 19^th^ century, the process of producing a spectrum from digital time series was largely accomplished by Fourier analysis. Fourier showed that any function showing certain minimal properties called the “Dirichlet Conditions” can be approximated by a harmonic series of orthogonal sine and cosine terms [6,7]. The series must have a finite number of maxima and minima, be defined at all points and not have an infinite number of discontinuities, conditions met by most data encountered in biology [12]. Here we take a function f(t) and approximate it with a Fourier series:

(1) $$f\left(t\right)≅a_{0}/2+a_{1}\sin t+a_{2}\sin2t+\ldots b_{1}\cos t+b_{2}\cos2t+\ldots$$

If our function consists of an ordered set of values x(*t*), then the “power” in the series is the ensemble average of the squared values. If the mean is zero, this is variance. The Fourier transform is an extension of a fit of the Fourier series and has the property that the coefficients approximate the spectrum of the power, meaning the power at each frequency for which a computation can be made (review: [5]). For a continuous series, we have:

(2) $$F\left(\omega \right)=\int_{-\infty }^{\infty }f\left(t\right)e^{-\mathrm{i\omega t}}\mathrm{dt}$$

**Figure 3:**
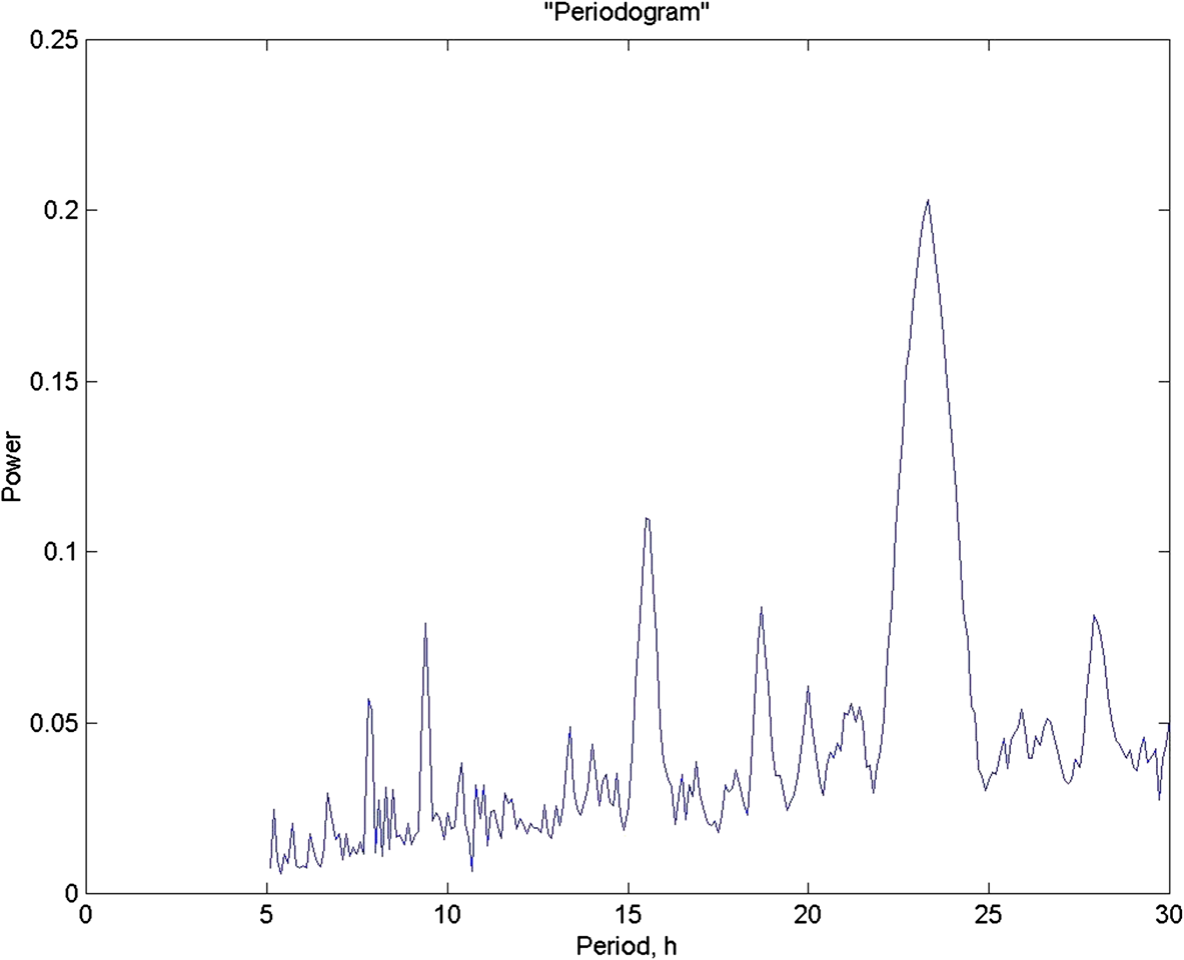
**This is the so-called Whitaker-Robinson “periodogram”, which is not the same as the true periodogram**_**ss **_**of Schuster.**

The exponential function consolidates the sine and cosine terms. *F*(*ω*) is the spectrum of the function, with *ω* being the angular velocity, or 2πf, where f is frequency. This process was carefully described by Schuster [13] and he termed it the “periodogram” of the function. This procedure should not be confused with the Whittaker-Robinson algorithm [14], improperly given the same name, which was largely discredited by Kendall on formal mathematical grounds [15]. (See [12] for further discussions and examples). Figure 3 depicts the Whitaker-Robinson “periodogram” for the data set.

**Figure 4:**
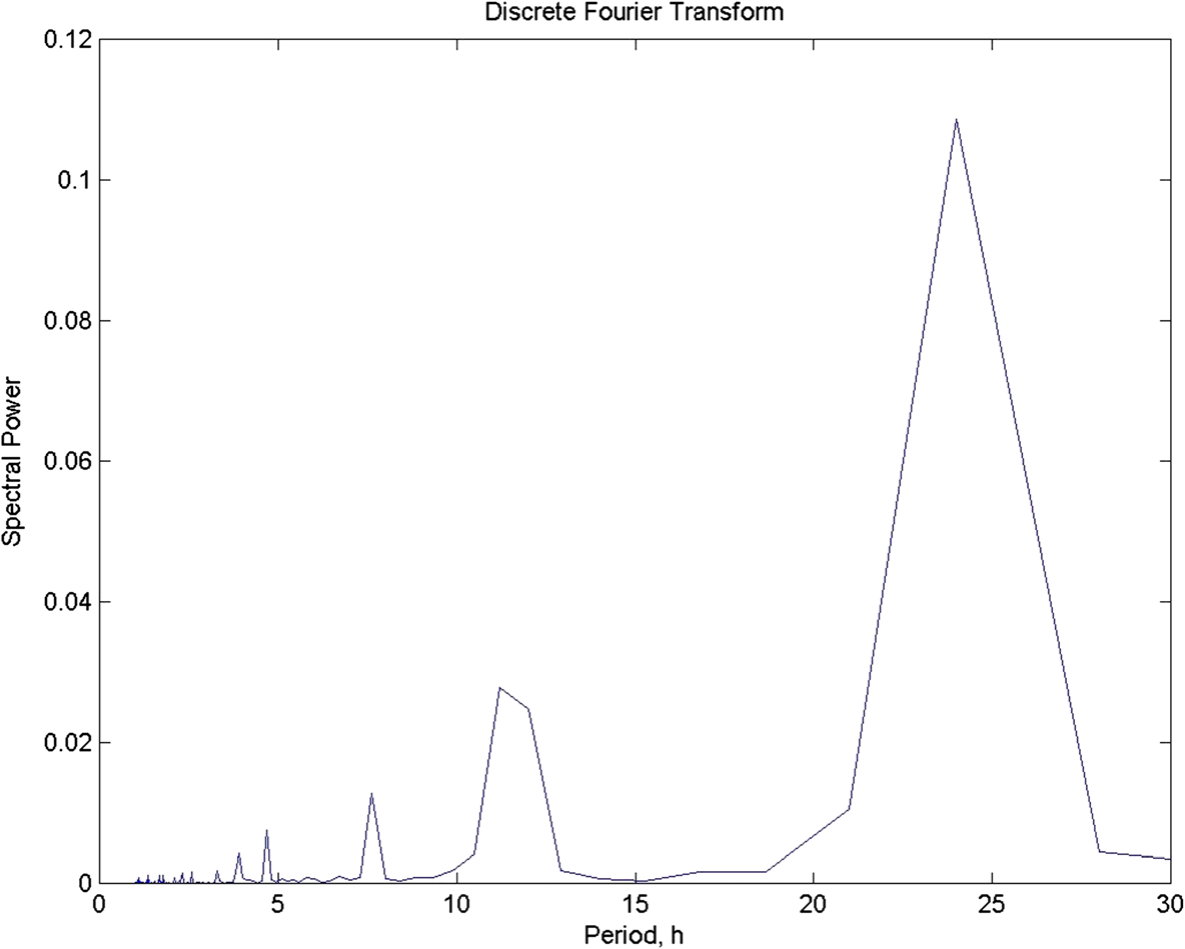
**The Discrete Fourier Transform of the test time series.** The period is calculated to be 22.4. Note in particular the paucity of spectral estimates in the crucial range between 20 and 30 hours. This would normally be corrected in more advanced Fourier Transform algorithms, but at a cost (see text).

Fourier analysis has undergone considerable development and sees a great deal of use in many fields, with chronobiology prominent among them; it has done yeoman service. If the spectrum is calculated directly from data sampled at intervals, it is termed the Discrete Fourier Transform or DFT. Fourier spectra are seldom computed directly from the raw data however, rather they are produced from either the autocovariance or autocorrelation functions. One argument for using the autocovariance function is that the output is equivalent to partitioning the variance in the signal by frequency and the area under the curve is the power (review: [5]). Figure 4 depicts the DFT of the test data set which is the most basic way to visualize the process. The period is reported as 22.4 h which contrasts with the known value of 23. The reason for this discrepancy is discussed below.

### Compromises inherent in Fourier analysis

Since the autcovariance and autocorrelation functions lose power with each pair of points lost, usually no more than one third of the data are used to compute correlation coefficients, adversely affecting the potential resolution in the spectrum. To alleviate this, the rest of the function is padded out with zeroes. This is an outright falsification of data points not in evidence, since there is no reason to suspect these data points would all be zeroes. An added problem occurs at the point where the zeroes abruptly start, since this abrupt discontinuity will cause artifactual peaks in the spectrum called “side lobes” owing to the Gibbs phenomenon [16]. To correct for this, the real data are blended into the zeros to soften the transition. Here, yet more actual data must be altered to allow for side lobe suppression. One further development that exacerbates these compromises is the Fast Fourier Transform, or FFT. In this algorithm, computational efficiency, and concomitantly speed of calculation, are increased by constraining the input series to consist of 2^N^ data points. Here again, the chances of a data set containing an integer power of 2 points are slim, and again, zeroes are added to pad the series out (Review [16]).

Further data corruption can occur when tighter spacing of spectral estimates is required. If the series is long, consisting of multiple cycles, this is usually not a problem. However, when short data sets are at hand, as is commonly the case with circadian rhythm work, there will be few cycles available. Fourier analysis is based on harmonics and these are constrained. In practice, this means that the spacing between estimates can be very wide. For a one-week long experiment with data sampled at half hour intervals (as in the test data) and analyzed using a simple DFT, spectral estimates are produced only for 22.4 and 24 h in the critical interval between 22 and 25 h. This leaves enormous gaps with little chance that the single estimate at 22.4 is even remotely close to the true period which is 23.0 h. Once again, it is straightforward to tighten up the interval between estimates, but once again, zeroes are added with further problems in using false data points for which there is no justification [16].

### Maximum entropy spectral analysis

In the late 1960’s, John Parker Burg developed a new method for producing a spectrum that tackles these problems [17,18]. It initially found acceptance in astrophysics and quickly spread to other fields. It began to be used for circadian rhythm work in the 1980s and is an excellent choice for a wide range of biological time series. This technique is called “Maximum Entropy Spectral Analysis” (MESA) [16-18]. MESA delivers the highest possible resolution, while eliminating side lobe problems. It is also extremely sensitive, as defined above. It is particularly useful in the short, noisy time series typical in biological systems [12,19,20].

The linchpin of this powerful technique is stochastic modeling. Time series evolve in time according to probabilistic laws and there are a number of models that can underlie such processes. One example is an autoregressive (AR) function (Review: [5]). The assumption is that the system moves forward in time as a function of previous values and a random noise component. The simplest example is a Markov process:

(3) $$X_{t}={\mathrm{aX}}_{t-1}+Z_{t}.$$

Where t is time, a is a coefficient derived from the data and Z_t_ is white noise [5]. This simplest process may be extended by going backwards in time to earlier and earlier values, with each weighted by a coefficient derived from the known observed values [5]:

(4) $$X_{t}={\mathrm{aX}}_{t-1}+{\mathrm{bX}}_{t-2}+\ldots +{\mathrm{cX}}_{t-p}+Z_{t}.$$

and, again, a, b, c,… are the model’s coefficients and p is the order of the filter. These coefficients form the prediction error filter (PEF) [21]. Crucially, it is possible to use the model to predict future values based on what is known of all the past values. In the case at hand, the analysis is functionally extending the autocorrelation function out to the needed number of values by prediction from those that can be reliably estimated [16]. Entropy, in information theory, is equivalent to ignorance. If one can formally calculate estimates that maximize ignorance, this means these values are the most honest based on what is known from data in hand and this is demonstrated through the calculus of variations [16-18]. A pile of zeros certainly does not fit this criterion. The spectrum is constructed from the coefficients as follows [17,18]:

(5) $$S\left(\omega \right)={P/\left|1-\sum_{k=1}^{p}a_{k}e^{-\mathrm{i\omega k}}\right|}^{2}$$

Where: *S*(ω) is spectral power as a function of angular velocity (see above), *P* is the power passed by the PEF, *p* is the order of the PEF and *a*_*k*_ is the set of PEF coefficients.

The algorithm commonly used by us and others calculates the filter in an iterative fashion and is based on the work of Anderson [22]. Each iteration extends the AR model by one. The number of coefficients in the prediction error filter employed to construct the spectrum is hence not fixed and requires some care in its choice. If a number that is too low is chosen, resolution and important detail can be lost. On the other side of the coin, if the number of coefficients is run up too high, there may be spurious peaks [21]. An objective method has been developed using the methods of Akaike [21], based on information theory. The filter length chosen is consistent with the most amount useful information that is being extracted as each iteration extends the length of the filter. This is used in the MESA software employed in our work, but we also commonly set a minimum filter length of about N/4 for biological rhythm analyses to ensure adequate representation of any long period cycles in the presence of noise, which can be considerable. N/3 is a good safe maximum [5,21].

**Figure 5:**
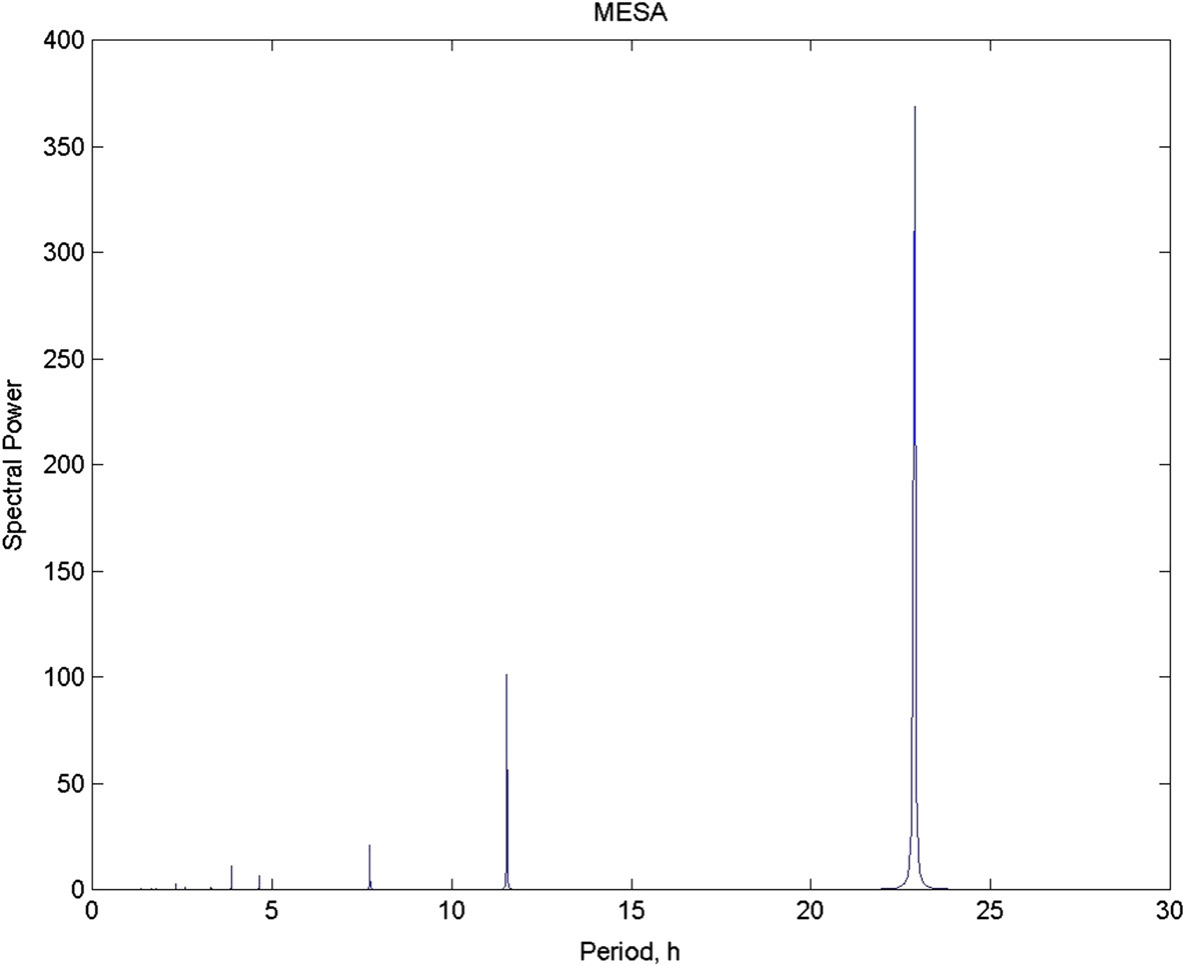
**This is the MESA spectrum with the coefficients upped to 32X.** The period reported is 22.88, compared to the known input of 23.0. The tiny discrepancy is likely a result of the 20% added noise in the signal.

MESA has proven itself superior to ordinary Fourier analysis as it does not produce artifacts from the various manipulations needed absent a model for the function and both resolution and sidelobe suppression are superior to standard Fourier analysis [16,23]. To show the difference between Fourier analysis and MESA in one critical area, it was noted above that the possible estimates that can be computed for period in Fourier analysis is constrained by the fact that these estimates can only be calculated for fixed values that are harmonics based on the length of the time series at hand. Longer series mean more tightly spaced estimates and this can be “faked” by adding zeros. MESA does not need to do this. Since the spectrum is extracted from an AR model, the spacing can be narrowed to any needed level. As an example, for the time series we have been working with, we have one week’s worth of data, sampled at half hour intervals. Note that shortening the bin size will have no effect on the spacing of the samples. Figure 5 shows the MESA for the test data set with the number of estimates increased by a factor of 32. Unlike the DFT, which had only 2 estimates in the interval between 22 and 25 h, MESA produced 60. Increasing MESA coefficients could go as high as needed, with the downside being a growing number of values that need plotting. Here 32X is more than sufficient to tease out a good estimate.

### Data conditioning

Biological signals can be notoriously non-stationary and noisy. This variation can take the form of linear or nonlinear trends in amplitude, variations in period and the waveform. As with any signal analysis system, MESA output can be improved by conditioning the signal. It should be noted, however, that MESA is robust in the face of such problems from the start. Incorporated into our MESA program is a detrending step which fits a line by regression and subtracts it. This eliminates linear trend and removes the mean. Mean removal is highly recommended, as this DC component can obscure the rhythmic one if it is excessive [12]. Removal of nonlinear trend can be accomplished by high-pass filtering by numerous methods and will not be discussed here as it is beyond the scope of this work. Low pass filtering to remove high frequency noise, however, is of considerable interest and is commonly done in preparation for spectral analysis (Review: [12,24]).

**Figure 6:**
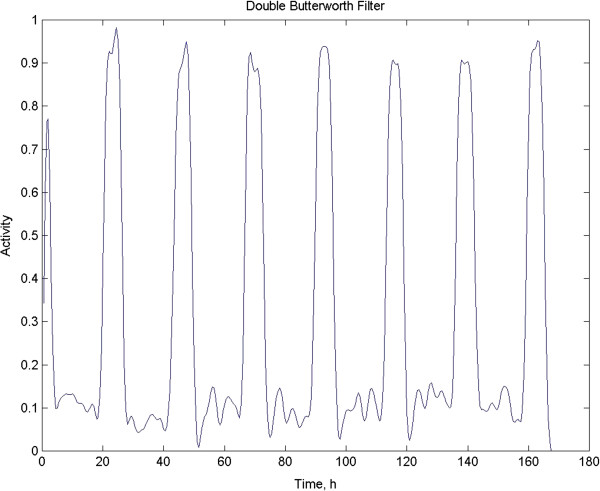
**This is the original data set after being filtered twice with a Butterworth recursive digital filter.** The second pass reverses the filter’s introduction of a four-hour phase delay owing to its recursive nature.

We have had excellent success with Butterworth recursive filters [9,12,24]. They are considered recursive because in addition to incorporating the original time series data into the moving filtering process, previously filtered values are used as well. Butterworth filters are highly accurate and reliable, and the cutoff frequency is sharp [9]. In Figure 6, the artificial test signal depicted in Figure 1 is shown after filtering with a two-pole low-pass Butterworth filter with a ~3 dB amplitude rolloff at the specified period of 4 h. The number of poles reflects the depth of the recursion [9]. The filter equation showing the recursion is:

(6) $$Y_{t}=\left(X_{t}+{2X}_{t-1}+X_{t-2}+{\mathrm{AY}}_{t-1}+{\mathrm{BY}}_{t-2}\right)/C$$

Where X_t_ is the original data series and Y_t_ is the output series. A and B are the filter coefficients: A = 9.656 and B = −3.4142. C is the “gain” or amplitude change of the filter and equals 10.2426. See [9,12,24] for a more detailed description of this filter. Owing to the recursion there is a 4-h phase delay in this example and this needs either to be made clear when the data are plotted or actually reversed. Reversal can easily be accomplished by running the filter in reverse. Since it is highly inadvisable to run a filter more than once to achieve additional smoothing before spectral analysis, as this will result in artifact [9], this reversal must only be done for display of simple plots for visualizing data. A single pass with the phase change is not an issue for MESA, since the attendant phase shift is of no consequence in this context. A second reversing pass with this filter actually resulted in a widening of the MESA peak (data not shown). After filtering, the RI (see above) is improved from 0.697 to 0.715.

### MESA at work

MESA has seen notable success since first being implemented for use in biological rhythms in the 1980s. MESA is useful for an extremely wide range of living oscillatory processes. It was instrumental in discovering the presence of ultradian rhythms in *Drosophila* locomotor activity rhythms early on, most remarkably in flies bearing the *per*^*0*^ and *per*^*-*^ mutations, which have no overt circadian periodicity [25,26]. These ultradian rhythms have been central in a competing hypothesis describing the mechanism of the circadian clock [26,27]. It has been used extensively in circadian rhythm work since that time. Given its superior resolving power, it settled an old dispute about the presence of lunar rhythmicity in physiological activity in marine organisms [9]. When applied in conjunction with powerful trend removal techniques, it was instrumental in teasing out the role of the gene *cryptochrome* (*cry*) in the fly clock system. Luciferase activity was monitored in antennae bearing either tim-luc or per-luc constructs and a central role for CRY protein in the peripheral antennal clock was established [28,29]. Ultradian and circadian rhythms were examined in premature infants (24–29 weeks) prior to their developing robust circadian periodicity enabling inferences on prenatal periodicity in normal pregnancies [30]. A cryptic human core body temperature of about one hour was elucidated [31]. Electroencephalography has yielded considerable information on ultradian periodicity in rats with MESA analysis combined with aggressive filtering enabling an incorporation of these high-frequency rhythms into models of sleep-wake dynamics [32]. A genetic component in strain differences among normal mice was discerned when locomotor activity was investigated with MESA, revealing robust ultradian components [33]. The presence of an endogenous vertical migration rhythm in Antarctic krill was verified [34]. Work on the cardiac pacemaker of the fly heart has benefitted from the use of MESA for measuring heart rate [35,36]. When combined with a novel preliminary Fourier treatment to alter the sampling structure, the presence of rhythmicity in the spacing of pulses in the *Drosophila* mating song was confirmed and it was shown to be under the control of the *period* gene [37].

In summary, Maximum Entropy Spectral Analysis has proven itself to be a highly useful and versatile tool for the investigation of periodic biological phenomena.

### Technical note

A full explanation of the mathematics underlying MESA and the ways in which algorithms have been implemented is beyond the scope of this paper. For those wishing to explore these topics in detail, the author recommends the following: For a good general introduction to the basic logic of MESA see Able’s review [16]; Burg’s original papers are the next step in seeing how the technique developed [17,18]; the very thorough paper by Ulrych and Bishop [21] should be sufficient to answer almost any mathematical question on the procedure and the algorithm used in our version of the technique, which we implemented in FORTRAN, is found in Andersen’s contribution [22]. Some of these papers, notably those of Burg himself, are difficult to locate. The compendium edited by D.G. Childers entitled “Modern Spectrum Analysis” (1978, Wiley) has all these recommended papers and many more that are on point.

All software used in this lab, including the FORTRAN source code, is available free of charge from the author: dowse@maine.edu. A step by step annotated guide in its use has been published by this author [38].

## Competing interests

The author declares that he has no competing interests.
